# Outcome of Infantile Malignant Solid Tumors: A Single-Center Experience

**DOI:** 10.3390/children12091276

**Published:** 2025-09-22

**Authors:** Burcu Tufan Taş, Nurşah Eker

**Affiliations:** Department of Pediatric Hematology and Oncology, Pendik Training and Research Hospital, Marmara University, Istanbul 34899, Turkey; nursah.eker@marmara.edu.tr

**Keywords:** infantile cancer, pediatric oncology, solid tumors, survival outcomes

## Abstract

**Background:** Malignant solid tumors diagnosed during the first year of life represent a rare but clinically significant subgroup of pediatric cancers. Their biological behavior, treatment responses, and prognosis differ substantially from tumors diagnosed in older children due to developmental immaturity and age-related therapeutic limitations. **Methods:** We retrospectively analyzed 88 infants diagnosed with malignant solid tumors before 12 months of age at a single tertiary center between March 2011 and March 2023. Demographic, clinical, pathological, and treatment data were collected. Overall survival (OS) was estimated by Kaplan–Meier analysis, and prognostic factors were evaluated using univariate and multivariate Cox regression models. **Results:** Of the 98 initially screened patients, 88 were eligible for analysis. The median age at diagnosis was 7 months, with a median follow-up of 42 months. The most common tumor locations were intra-abdominal (64.7%), brain (20.5%), and bone/soft tissue (12.5%). Neuroblastoma was the leading diagnosis (30.7%), with spontaneous regression observed in 29.6% of cases. Atypical teratoid rhabdoid tumor (ATRT) was the most frequent brain tumor (9.1%). The 5-year OS for the entire cohort was 78.3%. Brain tumors were associated with significantly higher mortality (HR 4.32, *p* = 0.01), while intra-abdominal tumors predicted improved survival (HR 0.31, *p* = 0.02). **Conclusions:** Infantile malignant solid tumors display heterogeneous clinical behavior and outcomes. While favorable results can be achieved in neuroblastoma and soft tissue sarcomas, brain tumors, particularly ATRT, remain a therapeutic challenge. Age-specific, risk-adapted treatment strategies and earlier detection are critical to improving survival and reducing long-term sequelae in this vulnerable population.

## 1. Introduction

Childhood cancers occurring within the first year of life are exceedingly rare. The same cancer types observed in this age group often exhibit distinct clinical and biological behavior compared to their presentation in older children. This difference is largely attributed to the immaturity and ongoing development of the infant organism, particularly during the neonatal period [1]. Early diagnosis and treatment of cancers in this early phase of life have a significant impact on both survival rates and comorbidities. Therefore, understanding the unique characteristics of this population is crucial [2].

The incidence of cancer varies between the neonatal (0–28 days) and infant (29 days–1 year) periods [1]. Neonatal tumors account for approximately 2% of all childhood malignancies [2]. During this period, genetically driven cancers and embryonal tumors are more prevalent [3], including neuroblastoma, retinoblastoma, nephroblastoma, and hepatoblastoma. In the infantile period, genetically based cancers are less frequently seen, and leukemia—though rarer than in older children—tends to follow a more aggressive course [4,5].

While embryonal tumors are more common in infancy and early childhood, carcinomas are rarely encountered. In several studies focusing on this age group, neuroblastoma has been the most frequent malignancy, followed by astrocytomas and medulloblastomas [6,7]. Solid tumors predominate in the neonatal and infantile periods; in one series, solid tumors accounted for 75% of all cancers diagnosed in this age group [8]. Treatment strategies often include close clinical and radiological monitoring, surgery, chemotherapy, monoclonal antibody therapy, and, rarely, multimodal regimens including radiotherapy. However, due to the patients’ young age, radiotherapy may not be administered as part of certain protocols or may be postponed until later in childhood, negatively impacting treatment success [5,9,10,11]. Therefore, tumors presenting in the first year of life should be considered as a separate subgroup within pediatric oncology that necessitates distinct diagnostic and therapeutic strategies [9].

In this study, we aimed to highlight the significance of cancers occurring in the infantile period by retrospectively evaluating the clinical characteristics, treatment responses, survival outcomes, and factors influencing survival in patients diagnosed before the age of one in our center.

## 2. Materials and Methods

Patients under the age of one who were diagnosed and followed with malignant solid tumors between March 2011 and March 2023 at Marmara University Faculty of Medicine, Pendik Training and Research Hospital, Department of Pediatric Hematology and Oncology, were retrospectively analyzed. Ethical approval for the study was obtained from the Ethics Committee of Marmara University (Approval number: 25-0424, dated 23 May 2025).

Patients were included after obtaining informed consent from their legal guardians. Data regarding age, sex, tumor location, presenting symptoms, pathological and radiological diagnoses, genetic findings (if any), surgical interventions, history of radiotherapy, chemotherapy protocols, clinical status, and last follow-up dates were retrieved from hospital records. Overall survival (OS) and prognostic factors affecting survival were analyzed.

Exclusion criteria were: patients diagnosed after one year of age, those with benign tumors before the age of one, hematologic malignancies, those who continued treatment or follow-up at other centers after diagnosis, those with irregular follow-up, and those without consent to participate.

The primary outcome of the study was overall survival (OS), defined as the time from diagnosis to death or last follow-up. Proportional hazards assumptions were tested using Schoenfeld residuals, and no major violations were detected. Missing data were handled by complete-case analysis. A 6-month age cut-point was selected based on prior literature and cohort distribution, as infants diagnosed after 6 months demonstrate distinct tumor biology and clinical outcomes. The impact of individual variables on survival was assessed using univariate and multivariate Cox regression analyses. A *p*-value of <0.05 was considered statistically significant.

Statistical analysis of the data obtained in this study was conducted using IBM SPSS Statistics for Windows, Version 23.0 (Armonk, NY, USA: IBM Corp.). Demographic and clinical characteristics of the patients were presented as frequencies, means, standard deviations, medians, and ranges.

The primary outcomes of the study were overall survival (OS) (defined as the time from diagnosis to death), progression, relapse, or last follow-up. Five-year OS, durations, and rates are presented in figures and tables. The impact of individual variables on survival was assessed using univariate and multivariate Cox regression analyses. A *p*-value of <0.05 was considered statistically significant.

## 3. Results

Between March 2011 and March 2023, a total of 98 patients diagnosed with malignant solid tumors under the age of one year were retrospectively reviewed. Nine patients with inaccessible medical records and one patient with a hematological malignancy were excluded from the analysis. One patient with a hematologic malignancy (leukemia) was excluded, as only patients with solid malignancies diagnosed before one year of age were eligible for this study. Complete records were unavailable for nine patients; seven continued their treatment at other centers, while one had incomplete records that precluded analysis. Among these nine excluded patients, four had stage IVS neuroblastoma, two had ATRT, one had non-metastatic Ewing sarcoma, and one had advanced-stage embryonal rhabdomyosarcoma. Of these, five were female and three were male, with a mean age at diagnosis of 8 months. The characteristics of excluded patients were not significantly different from those of the included patient. Ultimately, 88 infants with histopathologically confirmed solid tumors, diagnosed either at our institution or externally but treated and followed at our center, were analyzed. Following admission, all patients underwent radiological evaluation and definitive histopathological diagnosis. No additional biopsy sampling or cross-validation with external pathology review was performed during follow-up. Imaging assessments were conducted according to the respective treatment protocols.

The median age at diagnosis was 7 months (range: 1–12 months), and the median follow-up duration was 42 months (range: 1.0–141 months). The demographic characteristics of the patients are summarized in Table 1.

Based on the diagnostic distribution, 57 patients (64.7%) had intra-abdominal tumors, 18 (20.5%) had brain tumors, 11 (12.5%) had bone and soft tissue tumors, and 2 patients (2.3%) had other types of tumors.

Among intra-abdominal tumors, the most common diagnosis was neuroblastoma with 27 cases (30.7%), followed by germ cell tumors in 16 cases (18.2%), and Wilms tumor in 9 cases (10.2%). Less frequently encountered tumors included adrenocortical tumors and hepatoblastoma.

In the brain tumor group, the most common tumor was atypical teratoid/rhabdoid tumor (ATRT) (*n* = 8, 9.1%), followed by Langerhans cell histiocytosis (*n* = 4, 4.5%), medulloblastoma (*n* = 2, 2.3%), and optic glioma (*n* = 2, 2.3%). Less frequently observed tumors included pilocytic astrocytoma and other glial tumors.

In the bone and soft tissue tumor group, 11 cases (12.5%) were identified. The most common tumors in this group were rhabdomyosarcoma (*n* = 4, 4.5%) and infantile fibrosarcoma (*n* = 3, 3.4%), followed by Ewing sarcoma (*n* = 2, 2.3%). Other tumor types were less commonly seen (Table 2).

In the intra-abdominal tumor group, the most frequent presenting symptom was abdominal distension, observed in 17 patients (19.3%), followed by antenatally detected masses in 8 patients (9.1%). In the brain tumor group, the most common presenting symptoms were vomiting (*n* = 10, 55.5%) and ocular findings (*n* = 6, 6.8%). In the bone and soft tissue tumor group, limb swelling was the most common symptom, observed in 7 patients (63.6%) (Table 3).

Treatment protocols were determined based on tumor group, location, histological subtype, and stage, and included combinations of observation, surgery, chemotherapy, and radiotherapy. Specific protocols were as follows:

- Neuroblastoma: Turkish Pediatric Oncology Group (TPOG) protocol
- Brain tumors: Pediatric Oncology Group (POG 9233/34) protocol for children under 3 years of age (with published reference).
- Osteosarcoma: ECI (Etoposide, Cisplatin, Ifosfamide) and COSS-86/EURAMOS-1 protocols
- Ewing sarcoma: IE-VAC (Ifosfamide, Etoposide—Vincristine, Doxorubicin, Cyclophosphamide)
- Rhabdomyosarcoma: IRS-IV and IRS-V protocols
- Wilms tumor: National Wilms Tumor Study Group (NWTS) protocol
- Langerhans cell histiocytosis: LCH Society chemotherapy protocol

Metastatic sites included bone (*n* = 6), bone marrow (*n* = 8), lymph nodes (*n* = 2), and liver (*n* = 12).

- Bone (*n* = 6)
- Bone marrow (*n* = 8)
- Lymph nodes (*n* = 2)
- Liver (*n* = 12)

MYCN amplification was detected in 6 patients (22.2%) among those with stage IV disease. The staging distribution of the remaining patients was as follows:

- Stage IVS: 5 cases
- Stage III: 2 cases
- Stage II: 3 cases
- Stage I: 4 cases

Per protocol, in stage I and IVS disease, depending on the patient’s age and tumor volume, surgery alone or even observation may be sufficient. Among the 27 patients with neuroblastoma, 22 (81.4%) underwent diagnostic or therapeutic interventions:

- Total resection: 4 patients (14.8%)
- Subtotal resection: 3 patients (11.1%)
- Biopsy (open or trucut): 15 patients (55.5%)

Among the 8 patients with stage I and IVS disease, only 3 underwent total tumor resection; spontaneous remission was observed in the other 5 cases. Spontaneous regression was observed exclusively in patients with stage IVS neuroblastoma who were younger than 3 months of age at diagnosis.

Relapse occurred in 3 patients (11.1%), all of whom were in the stage IV group and all succumbed to progressive disease.

In the bone and soft tissue sarcoma group, the most common tumor was rhabdomyosarcoma, and all cases were diagnosed before 6 months of age. The most frequent subtype, diagnosed via biopsy, was embryonal rhabdomyosarcoma (*n* = 3), followed by alveolar rhabdomyosarcoma (*n* = 1). At the time of diagnosis, all patients underwent biopsy only. The extremities were the most common tumor site. Review of case distribution showed rhabdomyosarcoma in four patients, infantile fibrosarcoma in three patients, and Ewing sarcoma in two patients. Only 1 patient died—a case of metastatic embryonal rhabdomyosarcoma—due to progressive disease.

**Figure 1:**
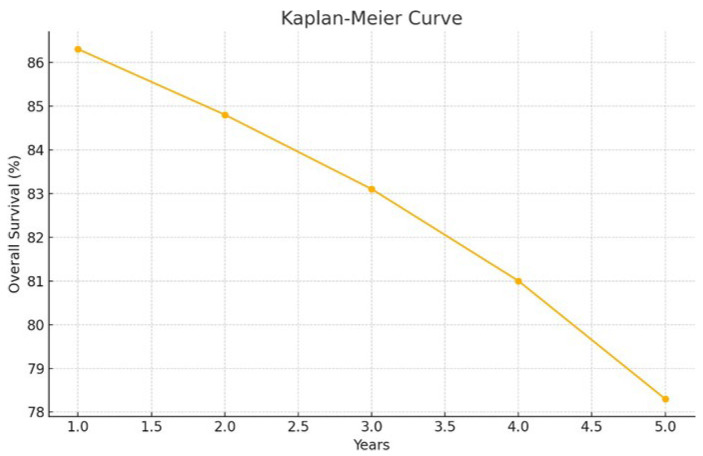
Curve Showing Overall Survival by Follow-Up Time.

The overall survival (OS) rates were: 1-year OS 86.3% (95% CI: 76.0–92.4), 2-year OS 84.8% (95% CI: 74.2–91.3), 3-year OS 83.1% (95% CI: 72.1–90.1), 4-year OS 81.0% (95% CI: 69.4–88.6), and 5-year OS 78.3% (95% CI: 65.5–86.8) (Figure 1).

- year OS: 86.3% (95% CI: 76.0–92.4)
- 2-year OS: 84.8% (95% CI: 74.2–91.3)
- 3-year OS: 83.1% (95% CI: 72.1–90.1)
- 4-year OS: 81.0% (95% CI: 69.4–88.6)
- 5-year OS: 78.3% (95% CI: 65.5–86.8) (Figure 1)

Prognostic factor analysis showed that age at diagnosis, sex, and treatment modality were not statistically significant predictors of survival. However, patients diagnosed at or after 6 months of age had a 3.606-fold increased mortality risk, though this was not statistically significant (Table 4). Patients who underwent complete or near-complete tumor resection had a lower mortality risk (HR = 0.412, *p* = 0.151), but this was also not statistically significant.

Variables such as sex and treatment modality were examined in univariable models but did not reach statistical significance and were not retained in the multivariable model.

Brain tumors were associated with a significantly higher risk of mortality compared to other tumor types (*p* = 0.005), whereas intra-abdominal tumors were associated with a significantly lower mortality risk (*p* = 0.008).

None of the patients were diagnosed with an underlying genetic disorder.

## 4. Discussion

Malignant solid tumors diagnosed during infancy constitute a distinct subset of pediatric cancers, exhibiting unique biological characteristics, clinical behavior, and therapeutic responses. Although they are rare, comprising approximately 2% of all pediatric malignancies [2], their management presents significant challenges due to age-related physiological immaturity, difficulty in early recognition, and limited evidence-based treatment protocols specific to this age group [1,3,4].

In our 12-year single-center cohort, neuroblastoma was the most commonly observed malignancy, consistent with previous studies that identify it as the leading solid tumor in infants [2,6,9]. Notably, 29.6% of our neuroblastoma patients experienced spontaneous regression, a phenomenon well-documented and particularly prevalent in localized tumors without MYCN amplification [12,13]. The NB95-S and NB97 prospective trials demonstrated that a “watch-and-wait” approach can be safely employed in selected patients, with spontaneous regression occurring in nearly half of the observed cases and excellent survival outcomes [12]. In our study, among 8 patients who showed regression, 5 received no surgical intervention and were managed with close observation. This supports the evolving paradigm of de-escalating treatment in biologically favorable neuroblastomas to avoid overtreatment and its long-term sequelae.

However, our data also highlight a considerable burden of advanced-stage disease at diagnosis. Nearly half (48.1%) of neuroblastoma cases presented with stage IV disease, and 22.2% harbored MYCN amplification, a known poor prognostic factor associated with aggressive clinical behavior [12,14]. These figures exceed those reported in countries with established neuroblastoma screening programs, where the prevalence of early-stage (I or IVS) disease is significantly higher [15,16]. For instance, Rubie et al. reported excellent outcomes with reduced-intensity therapy in infants with non-metastatic neuroblastoma lacking MYCN amplification [15]. In contrast, our higher rate of metastatic disease at diagnosis may reflect limitations in early detection, absence of screening initiatives, low socioeconomic status, and under-recognition of early tumor symptoms by caregivers [16,17,18]. This underscores the need to increase public and primary care awareness and potentially evaluate the feasibility of targeted screening strategies.

Our findings are also in agreement with previous reports on the anatomical distribution of neuroblastoma. The majority of cases involved the abdominal region, which aligns with previous epidemiologic studies, though thoracic and cervical tumors are more frequently observed in neonates [16]. In our cohort, complete or near-complete surgical resection was achieved in only a minority of neuroblastoma cases. While surgery remains a cornerstone of treatment in many pediatric solid tumors, its role in low-risk neuroblastoma is becoming more selective, especially given the potential for spontaneous regression and the risks associated with surgery in this age group [15,19].

In ATRT cases, radiotherapy was initiated immediately after surgery at a median age < 12 months, with 54 Gy to the cranial region and 36 Gy to the spinal field delivered in a total of 30 fractions using conformal techniques.

Surgical resection extent has consistently been shown to be a strong predictor of survival in pediatric CNS tumors [20,21,22,23]. In our cohort, gross total resection was only achieved in one of the eight ATRT cases, and incomplete resections were associated with poor outcomes. These results mirror findings from Toescu et al., who reported that even low-grade CNS tumors in infants often require multiple resections due to complex anatomical locations and high recurrence risk [24]. Our overall survival rate for CNS tumors was 61.1%, which is consistent with previous studies and population-based SEER data, which report 5-year survival rates of 46–67% for embryonal tumors in infancy [22,23].

Soft tissue and bone sarcomas comprised 12.5% of our cohort, with rhabdomyosarcoma (RMS) and infantile fibrosarcoma being the most frequent subtypes. RMS is the most common soft tissue sarcoma in infancy, and its management differs from that in older children due to the high sensitivity of developing tissues to radiation therapy [25,26]. As recommended by the Children’s Oncology Group (COG), our approach was to avoid or delay radiotherapy in these young patients to minimize long-term toxicity [27]. All RMS cases were managed with chemotherapy alone, and no local recurrences were observed. This finding is promising and aligns with the German CWS-81 study, which demonstrated favorable outcomes with age-adapted chemotherapy regimens [28].

In our series, infantile fibrosarcoma accounted for 3 cases and ranked as the second most common soft tissue sarcoma, aligning with previous reports. Histological subtype remains a crucial determinant of prognosis in this group, with non-alveolar RMS and fibrosarcoma generally having better outcomes than alveolar RMS. The overall survival rate for soft tissue sarcomas in our study was 81.8%, comparable to multicenter trials and institutional experiences.

From a broader perspective, the 5-year overall survival (OS) rate for all tumor types in our cohort was 78.3%, which is consistent with international reports ranging from 70–85% depending on tumor type, stage at diagnosis, and treatment availability [6,9]. Importantly, our multivariate analysis identified tumor type as the most significant prognostic factor: CNS tumors were associated with significantly higher mortality, while intra-abdominal tumors had more favorable outcomes. Notably, variables such as sex, age at diagnosis, and treatment modality were not statistically significant predictors of survival, although patients diagnosed after 6 months of age had a 3.6-fold higher mortality risk.

None of the patients in our cohort were diagnosed with an underlying genetic syndrome. While this may reflect true absence, it may also indicate underdiagnosis due to limited access to comprehensive genetic testing. It should be noted that comprehensive genetic testing was not consistently available throughout the study period, raising the possibility that predisposition syndromes may have been underdiagnosed.

Syndromic associations should be suspected in patients with multiple malignancies, developmental delay, or family history of cancer, as genetic predisposition syndromes like Beckwith-Wiedemann, Li-Fraumeni, or Gorlin syndrome have been associated with infantile tumors [5,11].

## 5. Conclusions

Malignant solid tumors diagnosed during infancy constitute a biologically and clinically unique subset of pediatric oncology. Our study demonstrates that neuroblastoma remains the most frequently encountered tumor type, often with a favorable prognosis due to its potential for spontaneous regression, particularly in low-stage, non-MYCN-amplified cases. However, delayed diagnosis and a high proportion of advanced-stage disease highlight the need for earlier detection strategies and increased awareness in underserved populations.

Central nervous system tumors, particularly atypical teratoid rhabdoid tumors, continue to pose significant therapeutic challenges due to their aggressive nature and limited resectability, resulting in poorer outcomes despite intensive treatment. In contrast, soft tissue sarcomas, especially rhabdomyosarcoma and infantile fibrosarcoma, can be effectively managed with chemotherapy-based approaches, with promising survival rates and reduced treatment-related toxicity when radiotherapy is deferred or avoided.

Our findings underscore the importance of age-specific, risk-adapted treatment strategies in infantile malignancies. Tailoring interventions to tumor biology, stage, and patient age is essential to optimizing survival while minimizing long-term sequelae. The major limitations of our study include its retrospective design, single-center setting, small subgroup sizes, protocol heterogeneity over the 2011–2023 study period, potential information bias due to inaccessible records, absence of standardized pathology re-review, and limited follow-up in some infants. Therefore, future multicenter, prospective studies are needed to refine treatment algorithms, explore the role of molecular profiling, and establish guidelines for the management of solid tumors in this uniquely vulnerable patient population.

## Figures and Tables

**Table 1:** The median age at diagnosis was 7 months (range: 1–12 months, IQR: 3–10), and the median follow-up duration was 42 months (range: 1–141 months, IQR: 20–67). The total number of patients was 88.

Variable	Value
Median Age at Diagnosis (months)	7
Median Follow-Up Duration (months)	42
Total Patients	88

**Table 2:** Tumor Types in the Infantile Period (corrected counts and percentages).

Tumor Type	Count	Percentage
Intra-abdominal	57	64.7
Neuroblastoma	27	30.7
Germ cell tumors	16	18.2
Wilms tumor	9	10.2
Brain	18	20.5
ATRT	8	9.1
Langerhans cell histiocytosis	4	4.5
Medulloblastoma	2	2.3
Optic glioma	2	2.3
others	2	2.3
Bone/Soft Tissue	11	12.5
Rhabdomyosarcoma	4	4.5
infantile fibrosarcoma	3	3.4
Ewing sarcoma	2	2.3
others	2	2.3
Others	2	2.3

**Table 3:** The most frequent presenting complaints were abdominal distension (19.3%), antenatally detected masses (9.1%), and ocular findings such as strabismus, nystagmus, and visual impairment (6.8%).

Presenting Complaint	Count	Percentage
Abdominal Distension	17	19.3
Antenatal Mass	8	9.1
Ocular Findings	6	6.8

**Table 4:** Prognostic Factors Affecting Overall Survival.

Factor	Univariable HR (95% CI)	*p*-Value	Multivariable HR (95% CI)	*p*-Value
Age ≥ 6 months	3.61 (0.85–15.4)	0.08	2.94 (0.65–13.2)	0.16
Complete resection	0.41 (0.12–1.41)	0.15	0.47 (0.13–1.68)	0.20
Brain tumor	4.85 (1.62–14.6)	0.005	4.32 (1.39–13.4)	0.01
Intra-abdominal tumor	0.28 (0.10–0.77)	0.008	0.31 (0.11–0.85)	0.02

## Data Availability

The original contributions presented in the study are included in the article; further inquiries can be directed to the corresponding author.

## References

[1] Reaman G.H., Bleyer A., Pizzo P.A., Poplack D.G. (2002). Infants and adolescents with cancer: Special considerations. Principles and Practice of Pediatric Oncology.

[2] Moore S.W., Satgé D., Sasco A.J., Zimmermann A., Plaschkes J. (2003). The epidemiology of neonatal tumours: Report of an international working group. Pediatr. Surg. Int..

[3] Moore S.W. (2013). Neonatal tumours. Pediatr. Surg..

[4] Biondi A., Cimino G., Pieters R., Pui C.H. (2000). Biological and therapeutic aspects of infant leukemia. Blood.

[5] Vormoor J., Chintagumpala M. (2012). Leukaemia and cancer in neonates. Semin. Fetal Neonatal Med..

[6] Lacour B., Goujon S., Guissou S., Guyot-Goubin A., Desmée S., Désandes E., Clavel J. (2014). Childhood cancer survival in France, 2000–2008. Eur. J. Cancer Prev..

[7] Alfaar A.S., Hassan W.M., Bakry M.S., Qaddoumi I. (2017). Neonates with cancer and causes of death: Lessons from 615 cases in the SEER databases. Cancer Med..

[8] Raciborska A., Bilska K., Węcławek-Tompol J., Ussowicz M., Pogorzała M., Janowska J., Rychłowska-Pruszyńska M., Rodriguez-Galindo C., Helwich E. (2016). Solid cancers in the premature and the newborn: Report of three national referral centers. Pediatr. Neonatol..

[9] Jin M., Tian Z., Xie Y., Zhang Z., Li M., Yu Y., Zhang W., Zhao J., Wang H., Zeng Q. (2020). Diagnosis and treatment of infantile malignant solid tumors in Beijing, China: A multicenter 10-year retrospective study. Pediatr. Investig..

[10] Isaacs H. (2009). Fetal brain tumors: A review of 154 cases. Am. J. Perinatol..

[11] Letterio J., Pateva I., Petrosiute A., Ahuja S. (2015). Hematologic and Oncologic Problems in the Fetus and Neonate.

[12] Hero B., Simon T., Spitz R., Ernestus K., Gnekow A.K., Scheel-Walter H.-G., Schwabe D., Schilling F.H., Benz-Bohm G., Berthold F. (2008). Localized infant neuroblastomas often show spontaneous regression: Results of the prospective trials NB95-S and NB97. J. Clin. Oncol..

[13] Brodeur G.M. (2018). Spontaneous regression of neuroblastoma. Cell Tissue Res..

[14] Carlsen N.L.T. (1990). How frequent is spontaneous remission of neuroblastomas? Implications for screening. Br. J. Cancer.

[15] Rubie H., de Bernardi B., Gerrard M., Canete A., Ladenstein R., Couturier J., Ambros P., Munzer C., Pearson A.D., Garaventa A. (2011). Excellent outcome with reduced treatment in infants with non-metastatic and unresectable neuroblastoma without MYCN amplification: Results of the prospective INES 99.1. J. Clin. Oncol..

[16] Sanchez V., Moreno C., Rosados P., Gutiérrez A., Jiménez J.I., Fos S., Valle J.C., Sada M. (1997). Neuroblastoma in children under one year of age. An. Esp. Pediatr..

[17] Orbach D., Sarnacki S., Brisse H.J., Gauthier-Villars M., Jarreau P.-H., Tsatsaris V., Baruchel A., Zerah M., Seigneur E., Peuchmaur M. (2013). Neonatal cancer. Lancet Oncol..

[18] Karnak I. (2016). Neuroblastoma: Current status from pediatric surgeon’s perspective. Turk. Assoc. Pediatr. Surg..

[19] Villarejo Ortega F., Aransay García A., Márquez Pérez T. (2016). Brain tumors in children. Pediatr. Integral..

[20] Toescu S.M., James G., Phipps K., Jeelani O., Thompson D., Hayward R., Aquilina K. (2019). Intracranial neoplasms in the first year of life: Results of a third cohort of patients from a single institution. Neurosurgery.

[21] Eker N., Tokuç G., Sarısaltık A., Dağçınar A., Gül D., Atasoy B.M., Tufan Taş B. (2024). Clinical factors, management, and outcomes of children under 3 years old with central nervous system tumors: Single-center experience. Child’s Nerv. Syst..

[22] Faltermeier C., Chai T., Syed S., Lau N., Elkaim L., Ibrahim G., Wang A., Weil A., Bendel A., Fallah A. (2019). Survival of infants ≤24 months of age with brain tumors: A population-based study using the SEER database. PLoS ONE.

[23] Loeb D.M., Thornton K., Shokek O. (2008). Pediatric soft tissue sarcomas. Pediatric soft tissue sarcomas. Surg. Clin. North Am..

[24] Jovaní Casano C., Canete Nieto A., Bermúdez Cortés M., Verdaguer Miralles A., Fernández Navarro J.M., Ferris Tortajada J., Castel V. (1998). Central nervous system tumors in children under 3 years of age. Esp. Pediatr..

[25] Ferrari A., Casanova M., Bisogno G., Zanetti I., Cecchetto G., De Bernardi B., Riccardi R., Tamaro P., Meazza C., Alaggio R. (2003). Rhabdomyosarcoma in infants younger than one year old. Cancer.

[26] Bradley J.A., Kayton M.L., Chi Y.Y., Hawkins D.S., Tian J., Breneman J., Wolden S.L., Walterhouse D., Rodeberg D.A., Donaldson S.S. (2019). Treatment Approach and Outcomes in Infants with Localized Rhabdomyosarcoma: A Report from the Soft Tissue Sarcoma Committee of the Children’s Oncology Group. Int. J. Radiat. Oncol. Biol. Phys..

[27] Koscielniak E., Harms D., Schmidt D., Ritter J., Keim M., Riehm H., Treuner J. (1989). Soft Tissue Sarcomas in Infants Younger than 1 Year of Age: A Report of the German Soft Tissue Sarcoma Study Group (CWS-81). Med. Pediatr. Oncol..

[28] Orbach D., Rey A., Oberlin O., de Toledo J.S., Terrier-Lacombe M., van Unnik A., Quintana E., Stevens M. (2005). Soft Tissue Sarcoma or Malignant Mesenchymal Tumors in the First Year of Life: Experience of the International Society of Pediatric Oncology (SIOP) Malignant Mesenchymal Tumor Committee. J. Clin. Oncol..

